# The association between the parental perception of the physical neighborhood environment and children’s location-specific physical activity

**DOI:** 10.1186/s12889-015-1937-5

**Published:** 2015-06-19

**Authors:** Sara D’Haese, Delfien Van Dyck, Ilse De Bourdeaudhuij, Benedicte Deforche, Greet Cardon

**Affiliations:** Faculty of Medicine and Health Sciences, Department of Movement and Sport Sciences, Ghent University, Watersportlaan 2, 9000 Ghent, Belgium; Research Foundation Flanders (FWO), Egmontstraat 5, 1000 Brussels, Belgium; Department of Human Biometrics and Biomechanics, Vrije Universiteit Brussel, Brussels, Belgium; Department of Public Health, Ghent University, De Pintelaan 185, 9000 Ghent, Belgium

**Keywords:** Activity, Children, Location, Neighborhood, NEWS

## Abstract

**Background:**

The relationship between children’s physical neighborhood environment and their physical activity, has been largely investigated. However in recent reviews, only a few significant and consistent direct associations between children’s physical neighborhood environment and their physical activity were found. This is possibly due to the fact that the location where children’s physical activity took place, is insufficiently specified. Therefore, this study aimed to investigate the association between parental perceived neighborhood characteristics and children’s physical activity in clearly defined environments.

**Methods:**

Children (9–12 years; n = 606) wore an Actigraph accelerometer for 7 days. Parents completed the parental version of the Neighborhood Environmental Walkability Scale questionnaire and reported on children’s physical activity in specific locations: physical activity in nearby streets and on sidewalks, physical activity in public recreation spaces and physical activity in the garden. Multilevel logistic regression analyses were conducted in MLwiN 2.30.

**Results:**

Children were more likely to be active in nearby streets and on sidewalks, if their parents perceived lower street connectivity (OR = 0.479; 95 % CI = 0.33 and 0.70), higher land use mix accessibility (OR = 1.704; 95 % CI = 1.25 and 2.33) and more crime safety (OR = 1.879; 95 % CI = 1.29 and 2.74). Children whose parents perceived higher presence of recreation facilities (OR = 1.618; CI = 1.23; 2.12) were more likely to be active in public recreation spaces. No environmental neighborhood variables were related to physical activity in the garden and overall moderate- to vigorous-intensity physical activity.

**Conclusions:**

The parental perceived physical neighborhood environment relates differently to physical activity in different locations. In order to develop effective interventions, it seems promising to further investigate the association between location-specific physical activity and specific neighborhood environmental correlates.

**Electronic supplementary material:**

The online version of this article (doi:10.1186/s12889-015-1937-5) contains supplementary material, which is available to authorized users.

## Background

It is recommended that children should engage in at least 60 min of moderate- to vigorous-intensity physical activity (=MVPA) per day [1, 2]. Being sufficiently physically active is associated with a decrease in cardiovascular risk factors [3], a reduction of the prevalence of obesity in children [4, 5] and may reduce the risk of osteoporosis at older age [6]. Despite the numerous health benefits of daily physical activity (=PA), there is evidence of decreasing PA levels in children [7]. Therefore, the promotion of PA during childhood has become an important public health aim [8] and interventions to promote children’s PA are necessary. To develop effective interventions to promote children’s PA, insight into PA determinants is necessary [9].

Recently, ecological models received increasing attention in health research. Ecological models posit that multiple levels of influence (e.g. intrapersonal, interpersonal, organizational, community, physical environmental and policy level) determine individual behavior [10]. These theories suggest a profound investigation of the physical neighborhood environment in order to create suitable interventions. The association between the physical neighborhood environment and PA in adults has been investigated often [11]. However, the associations between the physical neighborhood environment and PA in children are less understood than among adults [12, 13]. In recent reviews, only a few significant and consistent direct associations between children’s PA and the neighborhood environment were found [12, 14].

Different studies suggested that the weak associations between the neighborhood environment and PA, could be due to the fact that the location where children’s PA takes place, was insufficiently specified [14–17]. The predictive capacity of physical environmental correlates of PA may improve if PA is studied within clearly defined environments [16]. PA in a clearly defined environment (e.g. in the garden or in public recreation spaces) can be defined as “location-specific PA”.

The most investigated form of children’s location-specific PA is active transportation in the neighborhood [18, 19], but studies investigating environmental correlates of other location-specific physical activities are scarce. Only three studies were found, two from the USA and one from Australia, investigating perceived physical environmental correlates of other location-specific PA in children [15, 18, 20]. In the first study, proximity of recreation sites was positively related to PA in recreation sites [18]. In the second study, street connectivity was negatively and aesthetics were positively related to neighborhood PA; and crime safety and walk/cycle facilities were positively related to PA in public recreation spaces. This study clearly shows that the neighborhood physical environment relates differently to PA in different locations [15]. In Australia, positive associations were found between safety and living in a cul-de-sac, and play in the street [20].

More international evidence about the association between the neighborhood physical environment and children’s location-specific PA is necessary, as this can be very valuable for developing targeted interventions, aiming to increase children’s PA in specific locations (e.g. recreation facilities).

Therefore, this study aimed to investigate the association between parental perceived physical environmental characteristics of the neighborhood and children’s location-specific PA and overall MVPA. It was hypothesized that an activity friendly neighborhood for children would be positively associated with PA in the neighborhood and PA in public recreation spaces and would be negatively associated with PA in the garden. A second aim of the study was to determine the relation between parental perceived physical neighborhood environmental factors and children’s overall MVPA. It was hypothesized that the parental perceived neighborhood physical environment would not be related to children’s overall MVPA [16]. This was expected because large parts of children’s overall MVPA consist of PA at school [21] or organized PA at the sports club [22], and these domains of PA are probably not related to perceived physical neighborhood environmental characteristics.

## Methods

### Procedure

Data were collected between December 2011 and May 2013 as part of the Belgian Environmental Physical Activity Study in children (BEPAS-child). Principals (n = 46) from primary schools in Ghent were asked to participate. In total, 18 principals agreed and gave written informed consent (response rate = 34.6 %). Children and their parents from fourth, fifth and sixth grade (n = 994) were informed about the study and 606 parents gave written informed consent (response rate = 61.0 %). The Ethics Committee of the Ghent University Hospital approved the study.

### Measurements

#### Demographic variables

Children’s sex was derived from the children’s questionnaire and age from the parental questionnaire. One parent from each family was asked to fill out the questionnaire at home. There were 43 (7.0 %) parents that did not fill out the questionnaire after given informed consent. Educational attainment was used as a proxy measure for family SES (=socio-economic status), as educational attainment is easy to measure and is fairly stable beyond early adulthood, and higher levels of education are usually associated with better jobs, housing, neighborhoods, working conditions and higher incomes [23]. Parents were asked to report their own and their partner’s level of education (response options: primary school education, vocational secondary education, technical secondary education, general secondary education or art secondary education, college education or university education). Families were classified as high SES-families if the educational level of at least one parent was of a college or university level, if none of both parents reached a college or university degree, they were classified as low SES families [24–27].

#### PA in specific locations

Location-specific PA consisted of PA in public recreation spaces (inside or outside children’s neighborhood), PA in a garden (at home or elsewhere) and PA in nearby streets and on sidewalks, as these three locations have been identified as the locations where children mostly engage in active free play [28]. Parents responded to the question: “How often is your child active in/at following places during summer/spring?” Due to less favorable weather circumstances during winter/fall for outdoor PA in Belgium, only PA reported for summer/spring was included in this study. Response options were: never (=0), once a month or less (=1), every two weeks (=2), weekly (=4), 2 or 3 times a week (=10) and 4 times a week or more (=16). These response options were rescaled to indicate the number of times per month children were active in the specific locations. Test-retest reliability of location-specific PA variables ranged from ICC = 0.49 to 0.89 [29]. These measures were used in previous studies [15, 18].

*PA in public recreation spaces* was assessed with questions about frequency of activity in 1) a basketball court 2) a small public park or playground and 3) a large public park. These three items (Cronbach Alpha = 0.619) were summed and an average score was computed. It was not further specified whether the public recreation spaces were located inside our outside children’s neighborhood. *PA in the garden* was assessed with a single question: ‘How often is your child active in the garden during summer/spring?’ *PA in nearby streets and on sidewalks* was assessed with questions about frequency of activity 1) in a nearby cul-de-sac and 2) in a local street, sidewalk, or vacant lot. These two items (Cronbach Alpha = 0.621) were summed and an average score was computed. As these measures were ordinal and could not be treated as continuous variables, all these scales were dichotomized based on the median. An overview of the questions and the responses to these questions is shown in Additional file 1.

#### Overall MVPA

Children wore an ActiGraph™ GT1M, GT3X or GT3X+ accelerometer [30] (15 s epoch) during waking hours for 7 consecutive days. Strong agreement was found between these three activity monitors for measuring MVPA in children [30], making it acceptable to use different models within a given study. The accelerometer was worn on the right hip. Accelerometer data were screened, cleaned and scored using data-reduction software MeterPlus 4.2. Periods of 20 mins of consecutive zeros or more were defined as non-wear time [31]. Non-wear time activity diaries were provided to register activities for which the accelerometer was removed (e.g. swimming) and were used to replace the consecutive number of zeros by the corrected number of minutes MVPA [32]. A correction factor was used according to the type of activity to replace the missing accelerometer data. We multiplied the minutes of organized PA with 0.80, competition PA with 0.95 and curricular or leisure PA with 0.50 [32] to account for over-reporting in the activity diaries. MVPA (≥2296 counts/minute) was calculated using Evenson’s cutpoints [33, 34]. Children were included in the study if they had ≥2 weekdays with ≥10 h wearing time and ≥1 weekend day with ≥8 h wearing time [35]. Average minutes of MVPA per day were calculated and divided by the minutes of average daily wear time to obtain % of daily MVPA. %MVPA was dichotomized at the median.

#### Neighborhood variables

##### Perceived environmental factors

The parent version of the 'Neighborhood Environment Walkability Scale for Youth' (NEWS-Y) in Dutch was used to determine parental environmental perceptions of the neighborhood around participants’ houses, within a distance of ±1 kilometer (=10-15 min walking). The NEWS-Y determines the perceptions of residential density, the accessibility and diversity of land use mix, street connectivity, walk- and cycle infrastructure, aesthetics of the neighborhood and crime- and traffic safety. Internal consistency for all subscales and test-retest reliability of NEWS-Y for parents of 5–11 year old children were found to be acceptable [36].

All physical environmental variables were calculated following the NEWS-Y scoring guidelines with a higher score, denoting better PA conditions [37]. Parents also indicated whether they had a garden at their home or not. An overview of the questions and response options is presented in Additional file 2.

#### Analyses

Descriptive characteristics of the sample were analyzed using SPSS 20 (Released 2011. IBM SPSS Statistics for Windows, Version 20.0. Armonk, NY: IBM Corp.). Logistic regression analyses were conducted in MLwiN2.28 [38]. Multilevel modeling was used to take into account clustering of children (level 3) within classes (level 2) within schools (level 1). First, bivariate logistic regression analyses were conducted with perceived environment factors of the neighborhood as independent variable and location-specific PA (PA in public recreation spaces inside or outside the neighborhood, PA in the garden, PA in nearby streets and on sidewalks) and overall MVPA as dependent variables. Only children with a garden at their home were included in the analyses concerning garden PA. When a significant association was found in the bivariate analyses, this variable was entered into a multivariate logistic regression model. Model parameter estimates were obtained via Markov Chain Monte Carlo (MCMC) procedures [39].

Multicollinearity among independent variables was tested, by performing Pearsons' correlations. None of the variables was excluded as there were no correlations with r > 0.7 [40].

All logistic regression analyses were controlled for family SES, sex and age of the child. Odds ratios (=OR) and 95 % confidence intervals (=95 % CI) are reported.

## Results

### Descriptive characteristics of the sample

An overview of the descriptive characteristics is given in Table 1. Children were on average 10.9 ± 0.9 years old; 46.1 % were boys and 36.3 % had low family SES.

**Table 1:** Descriptive characteristics of the sample

	Overall sample^a^	Public recreation facilities^b^	Garden^b^	Neighborhood^b^
N	563	463	460	504
Child mean age (yr) (mean ± SD)	10.09 ± 0.9	10.90 ± 0.9	10.90 ± 0.9	10.92 ± 0.9
Gender				
Girls (%)	53.9	54.2	54.6	53.8
Boys (%)	46.1	45.8	45.4	46.2
Family SES				
Low family SES (%)	36.3	33.7	34.6	34.3
High family SES (%)	63.7	66.3	65.4	65.7
Public recreation spaces PA^c^				
≤1 time per month (%)	51.7	51.8		
>1 time per month (%)	48.3	48.2		
PA in the garden^c^				
≤10 times per month (%)	45.9		39.4	
>10 times per month(%)	54.1		60.6	
Neighborhood PA^c^				
≤1.5 times per month (%)	48.4			49.2
>1.5 times per month (%)	51.6			50.8
Having a garden at home				
Yes (%)	82.2		85.4	
No (%)	17.2		14.6	

*SD* standard deviation, *SES* socio-economic status, *PA* physical activity

^a^children whose parents filled out the questionnaire were included

^b^children included in the final model of the analyses concerning PA in public recreation facilities, in the garden and in the neighborhood

^c^cutpoint corresponds to the median

Of the sample, 48.3 % was active more than once a month in a public recreation space; 54.1 % was active more than 10 times a month in the garden and 51.6 % was active more than 1.5 times a month in their neighborhood.

### The association between the parental perceived physical neighborhood environment and children’s location-specific physical activity

#### Physical activity in public recreation spaces

The association between the parental perceived physical neighborhood environment and PA in public recreation spaces is described in Table 2. The bivariate analyses revealed that children whose parents perceived higher residential density (OR = 1.502; CI = 1.05; 2.14), land use mix accessibility (OR = 1.442; 95 % CI = 1.10; 1.89), presence of walk/cycle facilities (OR = 1.733; 95 % CI = 1.25; 2.41) and presence of recreation facilities (OR = 1.690; 95 % CI = 1.33; 2.15) were more active in public recreation spaces (in- or outside the neighborhood). In the final model, only the presence of recreation facilities remained significantly associated with PA in public recreation spaces (OR = 1.618; 95 % CI = 1.23; 2.12).

**Table 2:** Associations between the parental perceived physical environment and children’s physical activity in public recreation spaces inside our outside the neighborhood

	Bivariate associations^a^				Final model (n = 463)^b^		
	β ± SE	n	95%CI	OR	β ± SE	95%CI	OR
Land use mix diversity	0.155 ± 0.111	515	0.94; 1.45	1.17			
Residential density	**0.407 ± 0.181**	**473**	**1.05; 2.14**	**1.5**	0.255 ± 0.184	0.90; 1.85	1.29
Street connectivity	0.019 ± 0.184	518	0.71; 1.46	1.02			
Land use mix accessibility	**0.366 ± 0.139**	**521**	**1.10; 1.89**	**1.44**	0.139 ± 0.160	0.84; 1.57	1.15
Walk/cycle facilities	**0.550 ± 0.169**	**520**	**1.25; 2.41**	**1.73**	0.325 ± 0.192	0.95; 2.02	1.38
Aesthetics	0.067 ± 0.156	520	0.79; 1.45	1.07			
Traffic safety	0.234 ± 0.163	518	0.92; 1.74	1.26			
Crime safety	0.190 ± 0.152	518	0.90; 1.63	1.21			
Recreation facilities	**0.525 ± 0.123**	**508**	**1.33; 2.15**	**1.69**	**0.481 ± 0.139**	**1.23; 2.12**	**1.62**
Having a garden (ref = no)	0.138 ± 0.290	521	0.65; 2.03	1.15			

β multilevel bivariate linear regression coefficient, *n* number of children included in the analytical sample, *SE* standard error, *CI* confidence interval

Bold: *p* < 0.05

^a^Multilevel logistic regression analyses were controlled for age, sex and family SES

^b^Multilevel logistic regression analyses were controlled for age, sex and family SES and variables that were significantly related to public recreation spaces physical activity in the bivariate analyses

#### Physical activity in the garden

The association between the parental perceived physical neighborhood environment and PA in the garden is described in Table 3. In the bivariate analyses, children with a garden at home, were more likely to be active in the garden if their parents perceived lower land use mix diversity (OR = 0.762; 95 % CI = 0.60; 0.97) and lower residential density (OR = 0.625; 95 % CI = 0.44; 0.89). In the final model, none of the perceived environmental factors in the neighborhood was related to PA in the garden.

**Table 3:** Associations between the parental perceived physical environment and children’s physical activity in the garden

	Bivariate associations^a^				Final model (n = 393)^b^		
	β ± SE	n	95%CI	OR	β ± SE	95%CI	OR
Land use mix diversity	**−0.271 ± 0.123**	**393**	**0.60; 0.97**	**0.76**	−0.176 ± 0.133	0.65; 1.09	0.84
Residential density	**−0.470 ± 0.179**	**393**	**0.44; 0.89**	**0.63**	−0.376 ± 0.193	0.47; 1.00	0.69
Street connectivity	−0.336 ± 0.197	392	0.49; 1.05	0.72			
Land use mix accessibility	−0.163 ± 0.144	393	0.64; 1.13	0.85			
Walk/cycle facilities	−0.279 ± 0.174	393	0.54; 1.06	0.76			
Aesthetics	0.229 ± 0.177	393	0.89; 1.78	1.26			
Traffic safety	−0.194 ± 0.178	391	0.58; 1.17	0.82			
Crime safety	0.168 ± 0.171	391	0.85; 1.65	1.18			
Recreation facilities	−0.191 ± 0.129	389	0.64; 1.06	0.83			

β multilevel bivariate linear regression coefficient, *n* number of children included in the analytical sample (only children having a garden were included), *SE* standard error, *CI* confidence interval

Bold: *p* < 0.05

^a^Multilevel logistic regression analyses were controlled for age, sex and family SES

^b^Multilevel logistic regression analyses were controlled for age, sex and family SES and variables that were significantly related to garden physical activity in the bivariate analyses

#### Physical activity in nearby streets and on sidewalks

The association between the parental perceived physical neighborhood environment and PA nearby streets and on sidewalks is given in Table 4. In the bivariate analyses, children were more likely to be active in nearby streets and sidewalks, if their parents perceived lower street connectivity (OR = 0.494; 95 % CI = 0.35 and 0.69), higher land use mix accessibility (OR = 1.675; CI = 1.29 and 2.17), more traffic safety (OR = 1.948; 95 % CI = 1.42 and 2.67), more crime safety (OR = 2.354; 95 % CI = 1.73 and 3.20) and more recreation facilities (OR = 1.326; 95 % CI = 1.06 and 1.66). In the final model, street connectivity (OR = 0.479; 95 % CI = 0.33 and 0.70), land use mix accessibility (OR = 1.704; 95 % CI = 1.25 and 2.33) and crime safety (OR = 1.879; 95 % CI = 1.29 and 2.74) remained significantly associated with PA in nearby streets and on sidewalks.

**Table 4:** Associations between the parental perceived physical environment and children’s physical activity in their neighborhood

	Bivariate associations^a^				Final model (n = 504)^b^		
	β ± SE	n	95%CI	OR	β ± SE	95%CI	OR
Land use mix diversity	0.046 ± 0.103	515	0.86; 1.28	1.05			
Residential density	0.097 ± 0.157	472	0.81; 1.50	1.10			
Street connectivity	**−0.706 ± 0.172**	**519**	**0.35; 0.69**	**0.49**	**−0.736 ± 0.193**	**0.33; 0.70**	**0.47**
Land use mix accessibility	**0.516 ± 0.133**	**521**	**1.29; 2.17**	**1.68**	**0.533 ± 0.159**	**1.25; 2.33**	**1.70**
Walk/cycle facilities	−0.082 ± 0.146	520	0.69; 1.23	0.92			
Aesthetics	0.283 ± 0.146	520	1.00; 1.77	1.33			
Traffic safety	**0.667 ± 0.162**	**518**	**1.42; 2.67**	**1.95**	0.104 ± 0.211	0.73; 1.68	1.11
Crime safety	**0.856 ± 0.156**	**518**	**1.73; 3.20**	**2.35**	**0.631 ± 0.193**	**1.29; 2.74**	**1.88**
Recreation facilities	**0.282 ± 0.115**	**509**	**1.06; 1.66**	**1.33**	0.088 ± 0.128	0.85; 1.43	1.09
Having a garden (ref = no)	0.160 ± 0.258	521	0.71; 1.95	1.17			

β multilevel bivariate linear regression coefficient, *n* number of children included in the analytical sample, *SE* standard error, *CI* confidence interval

Bold: *p* < 0.05

^a^Multilevel logistic regression analyses were controlled for age, sex and family SES

^b^Multilevel logistic regression analyses were controlled for age, sex and family SES and variables that were significantly related to neighborhood physical activity in the bivariate analyses

#### The association between the parental perceived physical neighborhood environment and children’s overall MVPA

The association between the parental perceived physical neighborhood environment and children’s overall MVPA is described in Table 5. None of the parental perceived physical neighborhood environmental variables was related to children’s overall MVPA in the bivariate analyses, therefore, no multivariate analyses were conducted.

**Table 5:** Associations between the parental perceived physical environment and children’s objectively measured moderate- to vigorous-intensity physical activity

	Bivariate associations^a^			
	β ± SE	n	95%CI	OR
Land use mix diversity	−0.124 ± 0.131	437	0.68; 1.14	0.88
Residential density	0.143 ± 0.197	395	0.78; 1.70	1.15
Street connectivity	0.305 ± 0.207	437	0.90; 2.04	1.36
Land use mix accessibility	0.121 ± 0.160	439	0.82; 1.54	1.13
Walk/cycle facilities	0.236 ± 0.180	437	0.89; 1.80	1.27
Aesthetics	−0.140 ± 0.184	437	0.61; 1.25	0.87
Traffic safety	−0.035 ± 0.190	437	0.67; 1.40	0.97
Crime safety	−0.223 ± 0.180	437	0.56; 1.14	0.80
Recreation facilities	−0.236 ± 0.139	433	0.60; 1.04	0.79
Having a garden (ref = no)	0.240 ± 0.318	441	0.68; 2.37	1.27

β multilevel bivariate linear regression coefficient, *n* number of children included in the analytical sample, *SE* standard error, *CI* confidence interval

^a^Multilevel logistic regression analyses were controlled for age, sex and family SES

## Discussion

The main aim of this study was to investigate the association between parental perceived physical environmental characteristics of the neighborhood and children’s location-specific PA. Furthermore, the association between children’s physical neighborhood environment and their overall MVPA was investigated. As expected, physical neighborhood environmental correlates of children’s PA varied by PA location and perceived physical neighborhood characteristics were unrelated to children’s overall MVPA.

The presence of neighborhood recreation facilities was the most important condition for children to be active in public recreation spaces that were located in- or outside their neighborhood. This finding may imply that most reported PA in public recreation spaces took place in facilities that were located inside children’s neighborhood. All other perceived neighborhood characteristics were unrelated to PA in public recreation spaces that took place inside or outside the neighborhood. Proximity to recreational facilities may promote children’s activity in these facilities, as recreation facilities nearby children’s home are better accessible for children compared to recreational facilities outside the neighborhood. This indicates that intervention developers have to focus on the presence of these facilities, rather than focusing on e.g. the aesthetics along the road to these facilities, as aesthetics were unrelated to PA in recreation facilities. In a US study, small public parks, playgrounds, playfields/courts and large public parks were among the five most commonly used PA sites for children; and children were more active in smaller parks compared to larger parks [18]. This may indicate that providing sufficient public recreation spaces for children can possibly yield positive effects on children’s PA and that the presence of smaller parks nearby can be more effective in increasing PA than larger parks that are further away from children’s home. However, the present results should be interpreted with caution because reverse causality may be present. For example, it is possible that parents from children who are frequently active in a public recreation space are more aware of these facilities, compared to parents from children who are mostly active in the garden.

It is possible that not only the presence of recreation facilities is important to explain children’s PA in these facilities, but also the presence of features in the recreation facilities and their quality may play an important role in relation to children’s PA. For example, in an Australian study, park improvements (including the establishment of a walking track, a barbecue area, a playground,..) were positively associated with the number of park users, the number of people observed walking and being vigorously active [41]. Also in the US, park renovations appeared to increase visitation and overall PA in different age groups [42]. Future research is necessary to investigate if correlates of PA in public recreation spaces inside the neighborhood differ from correlates of PA in public recreation spaces outside the neighborhood.

Parental perceived land use mix accessibility and crime safety were positively associated with PA in nearby streets and on sidewalks. In another Belgian study that investigated the correlates of children’s active commuting to school, land use mix accessibility was also positively related to children’s active transport to school [43]. These findings may indicate that a neighborhood with a high perceived accessibility is important for children to be active in their neighborhood. The positive relation between crime safety and PA in nearby streets and on sidewalks was expected as safety concerns may cause parents to restrict their children to play outdoors [44]. Also in an Australian study, parental perceptions of safety were positively related to children’s play in their street [20]. A negative association was found between street connectivity and PA in nearby streets and on sidewalks. This negative association with street connectivity can be explained by the fact that a neighborhood with low connectivity is characterized by few intersections and more cul-de-sacs that reduce traffic volume, which results in safer places to play in the streets. The negative association between street connectivity and reported child activity in the neighborhood was also found in a US study [15] and shows that an activity friendly neighborhood for children differs from an activity friendly neighborhood for adults. In adult studies it has consistently been shown that a higher street connectivity is associated with more PA [45, 46]. The challenge for urban planners and policy makers is to develop a neighborhood in which people from different age groups are encouraged to be physically active. For example, this can be done by providing sufficient play space (e.g. small parks) in neighborhoods with a high street connectivity for walking and cycling.

In contrast to our hypothesis that an activity unfriendly neighborhood would be associated to more garden PA, but similar to the results of an Australian study [20], none of the perceived neighborhood environmental factors were related to children’s PA in the garden. Based on these findings, it is assumed that other factors (e.g. family environmental factors such as number of siblings, parental rules, parental encouragement) explain children’s PA in the garden and that intervening in children’s neighborhood environment will not influence children’s garden PA. However, further research is necessary as it is possible that specific garden characteristics (e.g. size of the garden) mediate the association between neighborhood characteristics and children’s garden PA.

These findings show that the physical neighborhood environment is mainly related to PA that actually takes place in children’s neighborhood (in nearby streets and on sidewalks) and is probably unrelated to PA in other contexts. This possibly explains the fact that the neighborhood physical environment was unrelated to children’s overall MVPA. As a large part of children’s overall MVPA takes place outside the neighborhood (e.g. in the sports club or at school) and only a small part of their overall PA takes place in the neighborhood or public recreation spaces, the influence of the neighborhood physical environment on children’s overall MVPA might be limited; whereas in adults, the neighborhood physical environment relates to overall MVPA in adults [47]. Also in an Australian study, the frequency children played in specific outdoor locations (i.e. their own street, their garden and in the park/playgrounds) was unrelated to overall MVPA [20]. However, in the present study, overall MVPA was measured during the school year. It is possible that the perceived neighborhood environment relates more strongly to overall MVPA during school vacations, because then children have less opportunities to be active at schools or in a sports clubs.

More insight into the location-specific PA correlates will be very informative for policy makers or urban planners, aiming to increase children’s PA levels in specific places (e.g. recreation facilities). Therefore, in future studies the use of GPS and/or SenseCams (wearable camera that takes photos automatically) in combination with accelerometers are promising tools for investigating the association between the environment and children’s location-specific PA. By using the combination of GPS and/or SenseCams and accelerometers, children’s PA can be exactly located in the neighborhood and data will not be biased by self-report. Also the use of activity diaries in combination with accelerometers might provide valuable information (e.g. where the activity took place) to investigate the relation between the perceived neighborhood environment and overall MVPA in specific locations. In future research, also the relation between the perceived neighborhood environment and objectively measured MVPA during vacation and other specific time periods (e.g. critical window MVPA (=after school until 6 pm)) should be investigated.

Strengths of this study were the use of the validated NEWS, the most commonly used questionnaire in the literature to assess environmental perceptions [48], the relatively large sample, the use of accelerometry to objectively determine MVPA and the use of parental perceptions of the physical environment. The cross-sectional study design is a limitation, as no causal relationships could be examined. Furthermore, no objective measures of location-specific PA were available which made it impossible to test the criterion validity of these measures. Also the neighborhood characteristics were measured by self-report. It is possible that correlated error might have influenced the association between the two self-reported measures (i.e. parental reported PA and parental reported neighborhood characteristics) to a small extent. Also the response rate of the principals was rather low, which may have limited the representativeness of the findings. For example, it is possible that the present results are not generalizable to children from schools with a lower SES, as participating schools had a slightly higher number of children with high SES compared to other schools in Ghent (e.g. 27.0 % of children’s mothers did not obtain a secondary education degree in participating schools versus 32.6 % in non-participating schools in Ghent). Besides, 7.0 % of the parents did not fill out the questionnaire after giving informed consent which can have resulted in a selection bias.

## Conclusions

This study suggests that the parental perceived physical neighborhood environment relates differently to children’s PA in specific locations. To promote children’s PA in nearby streets and on sidewalks, and in recreation facilities, easy-accessible neighborhoods to walk or cycle, and neighborhoods with a low street connectivity that are safe from crime should be designed, providing sufficient public open spaces for children to be active.

In contrast to adults, the neighborhood physical environment was not related to overall MVPA in children. This indicates that intervening in children’s neighborhood environment will probably not lead to an increase in their overall MVPA levels and that other factors are more important to explain children’s overall MVPA. The perceived neighborhood environment was only related to PA that actually took place in the neighborhood (e.g. in the streets, on sidewalks).

## Additional files

Additional file 1:
**Outline of the location specific physical activity questionnaire.**
Additional file 2:
**Outline of the NEWS-Y parent version.**

## Abbreviations

CI: Confidence interval
PA: Physical activity
MVPA: Moderate- to vigorous-intensity physical activity
