# Supplementary material for: The association between the parental perception of the physical neighborhood environment and children’s location-specific physical activity
Source: BMC Public Health. 2015 Jun 19;15:565. doi: 10.1186/s12889-015-1937-5 (PMC4474575; doi:10.1186/s12889-015-1937-5)
Supplement: Additional file 1: — Outline of the location specific physical activity questionnaire. [file 12889_2015_1937_MOESM1_ESM.docx]

## Additional file 1 – Outline of the location specific physical activity questionnaire

| **How often is your child active in/at the following places during summer/spring?** | **Never** | **Once a month or less** | **Every two weeks** | **Weekly** | **2 or 3 times a week** | **4 times a week or more** |
| --- | --- | --- | --- | --- | --- | --- |
| **PA in public recreation spaces** |  |  |  |  |  |  |
| A basketball court | 82.4 % | 7.0 % | 2.7 % | 2.5 % | 3.6 % | 1.9 % |
| A small public park or  playground | 22.8 % | 26.2 % | 18.0 % | 14.2 % | 8.2 % | 10.5 % |
| A large public park | 34.6 % | 32.2 % | 13.2 % | 10.4 % | 4.2 % | 5.3 % |
|  |  |  |  |  |  |  |
| **PA in the garden*** |  |  |  |  |  |  |
| In the garden | 4.1 % | 3.1 % | 2.5 % | 10.9 % | 18.8 % | 60.6 % |
|  |  |  |  |  |  |  |
| **PA in the neighborhood** |  |  |  |  |  |  |
| In a nearby cul-de-sac | 58.9 % | 6.5 % | 4.2 % | 8.2 % | 8.4 % | 13.9 % |
| In a local street, sidewalk, or vacant lot | 41.9 % | 8.3 % | 7.2 % | 11.7 % | 12.9 % | 18.0 % |

*only children included who have a garden at their home (n=393)
