# Supplementary material for: The association between the parental perception of the physical neighborhood environment and children’s location-specific physical activity
Source: BMC Public Health. 2015 Jun 19;15:565. doi: 10.1186/s12889-015-1937-5 (PMC4474575; doi:10.1186/s12889-015-1937-5)
Supplement: Additional file 2: — Outline of the NEWS-Y parent version. [file 12889_2015_1937_MOESM2_ESM.docx]

## Additional file 2 - Outline of the NEWS-Y parent version

| **Nr** | **Questions about the neighborhood** | **n** | **Parental mean**  **x±SD /5** | **% of respondents** | | | | |
| --- | --- | --- | --- | --- | --- | --- | --- | --- |
| **1** | **Residential density** | **496** | **2.2±0.7** | **None** | **A few** | **Half** | **Most** | **All** |
| *1a* | *How common are separate or stand alone one family homes?* | 518 | 2.6±1.3 | 25.1 | 26.6 | 16.8 | 22.6 | 8.9 |
| *1b* | *How common are connected townhouses or row houses?* | 535 | 3.2±1.1 | 7.7 | 24.5 | 23.9 | 33.8 | 10.7 |
| *1c* | *How common are apartment or condo buildings?* | 509 | 1.8±0.8 | 35.8 | 51.5 | 7.5 | 4.3 | 1.0 |
| **2** | **Land use mix accessibility** | **553** | **2.9±0.7** | **Strongly disagree** | **Somewhat disagree** | **Somewhat agree** | **Strongly agree** |  |
| *2a* | *From our home, it is easy to walk to school.* | 551 | 2.6±1.3 | 33.0 | 13.2 | 18.1 | 35.6 |  |
| *2b* | *There are many places where my child can walk to, alone or with someone else.* | 553 | 2.6±1.1 | 20.6 | 23.1 | 32.9 | 23.3 |  |
| *2c* | *From our home it is easy to walk to a bus or tram stop.* | 552 | 3.4±0.8 | 6.2 | 4.5 | 31.9 | 57.4 |  |
| *2d* | *It is easy to walk from one place to another (there is no motorway, railway or river).* | 550 | 3.0±1.0 | 11.3 | 15.5 | 37.1 | 36.2 |  |
| *2e* | *It is easy to walk to a playground or a park.* | 550 | 2.9±1.1 | 15.1 | 18.4 | 33.8 | 32.8 |  |
| **3** | **Land use mix diversity** | **546** | **3.4±0.9** | **> 30 min** | **21-30 min** | **11-20 min** | **6-10 min** | **1-5 min** |
|  | *How long should it take to walk to….* |  |  |  |  |  |  |  |
| *3a* | *convenience/small grocery store?* | 520 | 3.6±1.2 | 8.7 | 10 | 24.2 | 28.8 | 28.3 |
| *3b* | *supermarket?* | 529 | 2.8±1.4 | 22.7 | 16.8 | 27.2 | 18.9 | 14.4 |
| *3c* | *bakery?* | 537 | 3.8±1.1 | 5.2 | 7.6 | 22.9 | 32.6 | 31.7 |
| *3d* | *butcher’s* | 530 | 3.5±1.2 | 9.1 | 10.6 | 27.0 | 28.9 | 24.5 |
| *3e* | *newspaper stand?* | 534 | 3.6±1.2 | 7.1 | 11.0 | 25.8 | 29.6 | 26.4 |
| *3f* | *bank?* | 523 | 3.2±1.3 | 14.3 | 14.3 | 28.3 | 26.0 | 17.0 |
| *3g* | *library?* | 527 | 2.7±1.4 | 25.8 | 19.9 | 24.1 | 18.0 | 12.1 |
| *3h* | *my child’s school* | 533 | 2.8±1.4 | 29.1 | 11.6 | 23.3 | 22.3 | 13.7 |
| *3i* | *a bus or tram stop* | 535 | 4.4±0.9 | 1.9 | 2.1 | 8.0 | 27.7 | 60.4 |
| **4** | **Street connectivity** | **547** | **3.19±0.6** | **Strongly disagree** | **Somewhat disagree** | **Somewhat agree** | **Strongly agree** |  |
| *4a* | *The streets have many cul-de-sacs.* | 545 | 2.0±1.0 | 41.4 | 30.6 | 19.6 | 8.6 |  |
| *4b* | *There are many intersections.* | 543 | 2.9±0.9 | 9.8 | 20.1 | 43.5 | 26.7 |  |
| *4c* | *There are a lot of possibilities to walk or cycle from one place to another* | 543 | 2.6±0.9 | 11.6 | 29.3 | 45.3 | 13.8 |  |
| **5** | **Walk-/cycle-facilities** | **548** | **2.2±0.7** | **Strongly disagree** | **Somewhat disagree** | **Somewhat agree** | **Strongly agree** |  |
| *5a* | *There are sidewalks on most of the streets.* | 546 | 2.8±1.1 | 18.1 | 17.4 | 30.4 | 34.1 |  |
| *5b* | *There are bikeways on most of the streets.* | 547 | 1.8±0.9 | 42.0 | 37.7 | 14.1 | 6.2 |  |
| *5c* | *Bikeways are separated from the road by parked cars or green.* | 540 | 1.6±0.8 | 56.3 | 28.3 | 11.7 | 3.7 |  |
| *5d* | *Sidewalks are separated from the road by parked cars or green.* | 540 | 2.1±1.0 | 36.3 | 25.2 | 28.9 | 9.6 |  |
| *5e* | *There are bicycle sheds (at supermarkets, schools, bus stops...).* | 543 | 2.3±1.0 | 25.0 | 28.7 | 34.3 | 12.0 |  |
| **6** | **Neighborhood aesthetics** | **548** | **2.7±0.6** | **Strongly disagree** | **Somewhat disagree** | **Somewhat agree** | **Strongly agree** |  |
| *6a* | *There are many trees along the streets.* | 545 | 2.6±1.0 | 17.1 | 27.2 | 36.7 | 19.1 |  |
| *6b* | *There is little graffiti or garbage* | 545 | 3.0±0.9 | 9.7 | 11.6 | 43.3 | 35.4 |  |
| *6c* | *There is a beautiful scenery. (e.g. a beautiful landscape or view)* | 546 | 2.6±1.0 | 14.1 | 32.2 | 31.1 | 22.5 |  |
| *6d* | *There are many buildings/homes that are nice to look at.* | 545 | 2.8±0.9 | 9.4 | 23.1 | 50.3 | 17.2 |  |
| *6e* | *Public open spaces, playgrounds and parks are well maintained* | 543 | 2.7±0.9 | 10.5 | 27.1 | 46.4 | 16.0 |  |
| **7** | **Traffic safety** | **548** | **2.8±0.6** | **Strongly disagree** | **Somewhat disagree** | **Somewhat agree** | **Strongly agree** |  |
| *7a* | *Walking is dangerous because of the traffic.* | 546 | 2.6±0.9 | 11.2 | 37.9 | 32.6 | 18.3 |  |
| *7b* | *Cycling is dangerous because of the traffic.* | 546 | 2.9±0.9 | 6.4 | 26.4 | 39.4 | 27.8 |  |
| *7c* | *Cars usually drive slowly.* | 541 | 2.1±0.8 | 26.4 | 43.3 | 25.5 | 4.8 |  |
| *7d* | *Our streets are well-lighted at night.* | 539 | 2.7±0.7 | 5.9 | 25.6 | 57.0 | 11.5 |  |
| *7e* | *There are crosswalks and signals to help walkers cross busy streets.* | 545 | 2.6±0.9 | 14.3 | 27.0 | 47.9 | 10.8 |  |
| **8** | **Crime safety** | **548** | **2.4±0.7** | **Strongly disagree** | **Somewhat disagree** | **Somewhat agree** | **Strongly agree** |  |
| *8a* | *It is safe to play in the street* | 548 | 2.0±0.9 | 40.1 | 30.5 | 23.0 | 6.4 |  |
| *8b* | *There is a low crime rate.* | 539 | 2.8±0.8 | 9.8 | 15.8 | 57.0 | 17.4 |  |
| *8c* | *It is not necessary to be afraid of strangers when my child is walking down the street alone.* | 545 | 2.3±0.9 | 21.5 | 29.9 | 41.5 | 7.2 |  |
| *8d* | *It is not necessary to be afraid of strangers when my child is alone in a playground or a park.* | 538 | 2.2±0.9 | 23.8 | 38.1 | 33.1 | 5.0 |  |
| *8e* | *My bike is safe when I lock it.* | 543 | 2.6±0.9 | 13.6 | 23.8 | 48.8 | 13.8 |  |
| **9** | **Recreation facilities** | **537** | **2.7±0.9** | **> 30 min** | **21-30 min** | **11-20 min** | **6-10 min** | **1-5 min** |
|  | *How long should it take to walk to….* |  |  |  |  |  |  |  |
| *9a* | *an outside sports field?* | 515 | 2.9±1.4 | 23.1 | 17.1 | 23.9 | 19.6 | 16.3 |
| *9b* | *sea, lake, river or brook?* | 497 | 1.9±1.4 | 63.6 | 9.1 | 10.7 | 7.6 | 9.1 |
| *9c* | *walking- or cycling route?* | 487 | 3.2±1.5 | 22.2 | 11.7 | 16.8 | 22.8 | 26.5 |
| *9d* | *youth clubs* | 481 | 2.8±1.4 | 25.4 | 16.2 | 22.9 | 21.4 | 14.1 |
| *9e* | *swimming pool* | 518 | 2.0±1.2 | 48.5 | 19.1 | 18.3 | 9.1 | 5.0 |
| *9f* | *running track* | 490 | 1.9±1.2 | 55.7 | 16.7 | 13.9 | 8.0 | 5.7 |
| *9g* | *sports hall* | 505 | 2.3±1.2 | 38.6 | 19.6 | 24.8 | 11.7 | 5.3 |
| *9h* | *park or wood* | 510 | 3.0±1.5 | 23.5 | 12.4 | 19.8 | 25.1 | 19.2 |
| *9i* | *playground* | 515 | 3.2±1.4 | 19.4 | 10.3 | 21.4 | 27.0 | 21.9 |
| *9j* | *an open space to play (no park)* | 512 | 3.6±1.5 | 17.2 | 6.4 | 13.4 | 21.9 | 41.0 |

Residential density was calculated by following formula: score on question 1a + 12*score on question 1b + 25*score on question 1c and rescaled on a 5 point scale. All the other subscales were scored by taking the mean of the different question scores on a 5 point scale.

n = number of parents responding the questionnaire

x= mean

SD= standard deviation
